# Are maternal reflective functioning and attachment security associated with preadolescent mentalization?

**DOI:** 10.3389/fpsyg.2015.01134

**Published:** 2015-08-04

**Authors:** Anna Maria Rosso, Paola Viterbori, Alda M. Scopesi

**Affiliations:** Department of Education, Unit of Psychology, University of GenoaGenoa, Italy

**Keywords:** reflective functioning, mentalization, mental-state talk, attachment, adolescence

## Abstract

This study investigated the impact of maternal reflective functioning (RF) and attachment security on children’s mentalization. The Adult Attachment Interview (AAI) was administered to mothers in a sample of 41 mother–preadolescent dyads. AAI transcripts were rated in terms of the Berkeley AAI System (Main and Goldwyn, 1998) and the Reflective Functioning Scale (RFS; Fonagy et al., 1998). Preadolescent mentalization was assessed using a semi-structured interview adapted from O’Connor and Hirsch (1999) and also by analyzing mental-state talk produced during an autobiographical interview. Relationships between maternal RF and children’s mentalization were analyzed, with consideration given to the different RFS markers and references to positive, negative, and mixed-ambivalent mental states. Children’s mentalization was positively correlated with the mother’s RF, particularly the mother’s ability to mentalize negative or mixed-ambivalent mental states. No significant differences in mentalization were observed between children of secure and insecure mothers.

## Introduction

The present study focuses on the relationship of the mother’s reflective functioning (RF) and her attachment security with her preadolescent children’s ability to mentalize.

Mentalizing refers to the capacity to perceive and understand oneself and others in terms of mental states (feelings, beliefs, intentions, and desires) as well as the ability to reason about one’s own and others’ behavior in terms of mental states (Fonagy et al., 1998).

Fonagy et al. (1998) introduced the concept of mentalization, operationalized as RF in the wake of studies conducted on the “theory of mind” (ToM) within the domain of cognitive psychology (e.g., Leslie, 1987; Perner, 1991). His research and theoretical assumptions focused mainly on the development of mentalization and individual differences, expanding the concept beyond false beliefs, autism, and childhood (e.g., Baron-Cohen et al., 1985; Perner et al., 1989).

According to Fonagy et al. (1998), mentalization is necessary for good social adjustment because it allows one to represent causal mental states, discriminate internal, and external realities, infer other’s mental states from behavioral and contextual clues and regulate behavior and emotional experience.

In this perspective, mentalization is a protective factor for the individual and one’s social development. Consistent with this standpoint, many studies have found deficits in mentalization ability in psychopathological conditions (Bateman and Fonagy, 2004; Fonagy and Bateman, 2008; Rothschild-Yakar et al., 2010; Fonagy et al., 2011; Sharp et al., 2011). Similarly, satisfactory mentalization ability was found to be protective against the development of psychopathology (Sharp et al., 2006; Ostler et al., 2010).

The development of mentalization critically depends upon interpersonal experiences and specifically upon interactions with more mature minds, assuming these interactions are benign, reflective, and sufficiently attuned (Fonagy, 2006). Actually, mentalization deficits have been found in individuals who experienced abuse. For instance, abused children showed developmental delay in emotion-understanding processes (Frodi and Smetana, 1984; Smith and Walden, 1999) and in the performance of ToM tasks (Cicchetti et al., 2003; Pears and Fischler, 2005), independent of their intellectual level and socioeconomic condition. Likewise, traumatized children found it difficult to learn an emotional lexicon (Beeghly and Cicchetti, 1994) just as adults who were abused as children exhibited deficits in the ability to recognize facial expressions (Fonagy et al., 2003).

Currently, it is thought that maternal mentalization allows children to develop their mentalization capacity through the mother’s ability to regulate the child’s affective state, especially in moments of increased arousal. Mothers who respond to their children’s affective displays with contingent marked affective displays of their own allow their babies to modulate their own affective states; this process is thought to be especially important in the modulation of negative emotions (Fonagy et al., 2002) because it permits the child to not activate an attachment system that would deactivate the mentalization system (Fonagy, 2006; Fonagy and Target, 2008). Recent neuroimaging studies have supported this hypothesis (Bartels and Zeki, 2000, 2004), demonstrating that the activation of attachment system-related brain areas deactivates mentalization-related brain areas.

Mothers who are able to mentalize the affective states of their children help them to develop their own abilities to regulate emotions, which in turn permit them to process emotional stimuli without defensively avoiding them or feeling overwhelmed. Maternal mentalization allows the child to adapt to affective states without being destabilized and to experience and recognize the affect that he/she feels. This experience permits the development of the child’s ability to know the subjective meaning of his/her feelings beyond a mere intellectual understanding. It also promotes the ability to acquire “mentalized affectivity,” which would allow the child to feel thoughts and think about feelings (Jurist, 2005).

Although many studies have investigated the relationship between the mother’s mentalizing ability and the child’s attachment security, very few empirical studies have addressed maternal influences on the child’s actual mentalizing ability. The latter focused on: (1) the relationship between the mother’s attachment security and the child’s mentalization, and (2) the relationship between the mother’s mentalization and the child’s ability to understand emotions and solve ToM tasks.

The mother’s security with respect to her own childhood experiences has been shown to be predictive of her child’s ability to recognize painful feelings and face difficult situations (Steele et al., 2002), understand emotions, especially negative ones (Laible and Thompson, 1998; Steele et al., 1999, 2003, 2008), and solve false-belief tasks (Fonagy et al., 1997).

However, only a few empirical studies, generally limited to preschool-aged children, have investigated the relationship between the mother’s and child’s mentalization abilities. Meins et al. (2002, 2003), for example, observed a positive correlation between a mother’s “mind-mindedness,” defined as the mother’s ability to interpret the child’s internal states accurately, and her 4-year-old child’s performance on a battery of ToM tasks. Other studies have shown positive correlations between a child’s performance on Harris (1989) belief-desire ToM tasks, a maternal proclivity to describe children in mentalistic terms (Meins et al., 1998), and maternal RF (Steele and Steele, 2008).

Some studies have focused on the maternal tendency to face painful emotional states. Consistent with Fonagy et al.’s (2002) hypothesis, the maternal ability to talk about negative emotions has been shown to be predictive of the ability to understand emotions (Dunn and Brown, 2001) and of the early acquisition of ToM (Hughes and Dunn, 2002). A child’s tendency to avoid recognizing and facing negative emotions has been negatively correlated with the maternal ability to understand the child’s mind (Sharp et al., 2006), and more frequent maternal mental-state talk about positive feelings was found to be associated with less maternal RF and sensitivity to child needs (Borelli et al., 2012). Another study demonstrated that family dialog involving negative emotions was predictive of better performance on tests of understanding emotions (Dunn and Brown, 2001). Regarding maternal attachment patterns, an association between dismissive attachment and deficits in processing negative emotions was observed. Specifically, recent neuroimaging studies demonstrated that dismissive mothers are not able to process sadness (Strathearn et al., 2009) and inhibit negative affective responses (Leckman et al., 2004; Strathearn, 2006; Crittenden, 2008). Therefore, these mothers cannot mirror these same emotions in their children.

Up to now the relationship between maternal and childhood mentalization during early adolescence is understudied in that most research has focused on pre-school children. To the best of our knowledge, only one study (Benbassat and Priel, 2012) has investigated the role of parental RF on adolescent adjustment; it found that parental RF correlated with adolescent RF and social competence in a sample of adolescents aged 14–18 years. The impact of maternal attachment security on child mentalizing ability has also been investigated only in childhood.

During early adolescence, mentalizing abilities increase (Choudhury et al., 2006; Dumontheil et al., 2010; Goldstein and Winner, 2012; Valle et al., 2015) concurrently with other cognitive changes, such as understanding the concepts of possibility and logical necessity (Piéraut-Le Bonniec, 1980). Bosco et al. (2014) noted that the development of ToM is particularly consistent between ages 11 and 13 years. Furthermore, preadolescence is an important turning point in the development of narrative thinking (Bruner, 1986). Between the ages of 10 and 12, there is a clear shift in plot structure and in the interpretative understanding of human actions (Genereux and McKeough, 2007).

Investigations into the relationships among maternal mentalization, attachment security and mentalization in preadolescent children could aid in the design of preventive and therapeutic actions focused on the parent–child relationship during this under-considered developmental period. The importance of parental sensitivity during preadolescence is also frequently underestimated even though the early adolescent really needs to be understood as a feeling and thinking individual in the face of multifaceted developmental challenges, and impaired mentalization in both the parent and the child may be a serious risk factor for the emergence of family conflict, behavior disorders in childhood, and psychopathology.

Recent studies found that during adolescence mentalization is a protective factor against the emergence of proactive aggression (Taubner et al., 2013) and eating disordered behavior (Rothschild-Yakar et al., 2010, 2013). Other studies have shown that mentalization deficits in adolescence have an impact on BPD symptoms through the mediating role of emotion dysregulation (Sharp et al., 2011; Ha et al., 2013).

The goal of this study was to investigate the relationship between maternal reflecting functioning, maternal attachment security and the mentalization abilities of their preadolescent children.

A previous study on the same dyads (Scopesi et al., 2015) analyzed the relationship between the mental state talk of mothers and their preadolescent children, finding a significant role of maternal RF in children’s mental state talk. In this study, this relationship was further analyzed by taking into account not only the overall mothers’ RF but also their specific markers of mentalizing as well as the emotional context in which they mentalized. In particular, four markers of RF were considered, namely, “Awareness of the nature of mental states” (marker A), “Explicit effort to tease out mental states’ underlying behavior” (marker B), “Recognizing developmental aspects of mental states” (marker C) and “Mental states in relation to the interviewer” (marker D), (Fonagy et al., 1998). In addition, as the examined literature revealed a crucial role of the maternal ability to mentalize negative emotions, we differentiated maternal mentalization of positive, negative and mixed-ambivalent mental states, hypothesizing that mothers who can mentalize negative and mixed-ambivalent mental states have children who are more competent mentalizers themselves.

Concerning maternal attachment patterns, this study took into account both a categorical classification and a dimensional approach, considering both the Adult Attachment Interview (AAI; Main and Goldwyn, 1998) overall classification and the scores reported by the mothers on AAI’s state-of-mind subscales. It was expected that mother’s RF would significantly correlate with the mentalization ability in their children whereas the association between maternal attachment security and childhood mentalization would be less significant; however, it was hypothesized that a maternal idealizing or derogatory state of mind regarding attachment would be associated with less mentalizing in children.

## Materials and Methods

### Participants

A total of 41 mother–child dyads agreed to participate in this study. The children included 15 females and 26 males, aged 12.3–12.11 years, who were attending the state school. Subjects were from the working and middle socioeconomic classes. Most subjects were from intact families (75.6%), and most children were firstborns (65.9%). No subject suffered from a learning disorder or any other psychological condition as reported by the mothers and teachers. Only 59% of parents gave consent for their children to be administered the Wechsler Intelligence Scale for Children (WISC)-III. All of the children’s IQ results were within normal standards (range = 99–140; *M* = 115.88; SD = 11.22).

All participants were born in Italy from Italian parents, and Italian was the first or sole language for all of them. The mothers ranged in age from 37 to 53 years (*M* = 43.39; SD = 4.66). Their education level was between 8 and 23 years (*M* = 13.51, SD = 3.5), with 34.1% reporting 16 or more years of education, 46.4% reporting 13–15 years of education, and 19.5% reporting fewer than 12 years of education. They each had between 1 and 5 children (*M* = 1.98; SD = 0.82). Among the 41 mothers, 39 women (95.1%) reported working full-time outside the home.

### Measures

#### Maternal Attachment Pattern

The AAI (Main and Goldwyn, 1998) was administered to mothers to assess their attachment pattern. This well-established and widely validated semi-structured interview takes about an hour and includes 18 questions concerning childhood experiences of attachment. The coding system allows the classification of the attachment state of mind into five categories: secure (F), dismissive (Ds), preoccupied (E), unresolved with respect to past loss or trauma (U), Cannot Classify (CC).

Because of the sample size in this study, analyses based on attachment pattern classifications were conducted using the standard secure-versus-insecure (Dismissive, Preoccupied, Unresolved, and Cannot Classify) categorization. Moreover, as suggested by Bakermans-Kranenburg and van IJzendoorn (2009), a dimensional approach to AAI data was used in addition to the categorical system. To explore the relationships between specific dimensions of maternal attachment representations and the mentalizing abilities of their children, this study took into account the following continuous rating scales of the AAI transcripts: “Idealization regarding mother,” “Idealization regarding father,” “Derogation regarding mother,” “Derogation regarding father,” “Overall derogation of attachment,” “Insistence on lack of recall,” “Involving anger,” “Passivity of thought processes,” and “Coherence of the transcript.”

All of the AAIs were coded in terms of the Berkeley AAI System (Main and Goldwyn, 1998) by the first author. Ten randomly selected AAIs were then re-coded by an independent coder. The resulting inter-rater reliability was excellent (Cohen’s *k* = 0.84).

#### Maternal Reflective Functioning

The Reflective Functioning Scale (RFS) developed by Fonagy et al. (1998) was applied to the AAI transcripts to evaluate the mothers’ RF. The RFS was designed to use the AAI narratives to evaluate the capacity for mentalization, namely, “the capacity to perceive and understand oneself and others in terms of mental states” and “the capacity to reason about one’s own and others’ behavior in terms of mental states” (Fonagy et al., 1998, p.7).

In the AAI protocol, some questions require a RF (e.g., “Why do you think your parents behaved as they did during your childhood?”), while others merely permit it (e.g., “Could you describe your first separation from your parents?”).

According to the RFS scoring guidelines, there are four markers of reflecting functioning: “Awareness of the nature of mental states” (marker A), “Explicit effort to tease out mental states’ underlying behavior” (marker B), “Recognizing developmental aspects of mental states” (marker C), and “Mental states in relation to the interviewer” (marker D). The following statements are illustrations of RF. For instance, awareness of the nature of mental states (marker A) could be evident in the following sentence: “My mother often seemed happy, but I think that probably she sometimes would not show to us her concerns,” in which the subject shows that he is aware of the opaqueness of the mental states and that mental states are susceptible to disguise. In addition, the explicit effort to tease out the mental states underlying behavior (marker B) is exemplified in this statement: “I screamed because I felt awfully helpless, I was not able to think what to do, I felt really vulnerable!” An example of marker C is “When I was young I felt my brother was a nasty boy, now I think he was a very sad child” (“Recognizing developmental aspects of mental states”) while “I think that it can be painful for you to have to hear this sad story!” illustrates marker D (“Mental states in relation to the interviewer”).

After rating each identified passage of the AAI, an overall classification is assigned to the interview considered as a whole, ranging from –1 (negative RF) to 9 (exceptional RF).

In this study, the following variables were taken into account: the RFS overall score, the frequency of each of the four markers of RF, and the frequency of the references to positive, negative and mixed-ambivalent mental states in the context of RF.

The following is an example of mentalizing about mixed-ambivalent mental states: “When I grew up I became rather confused about the way I felt about my mother: When I was a child I felt she was my father’s victim; once I became a teenager I noted that their relationship was much more complicated than I thought earlier. Sometimes I thought she provoked his anger; for instance, when she went out without telling him when she should come back; sometimes she came back the day after, so I realized that she had a love affair and I did not know if she was right or wrong. Sometimes I was sympathetic with her; sometimes I felt really cross.”

Validation studies of the RFS (Fonagy et al., 1998) have documented discriminant and predictive validity, good inter-rater reliability, low correlations with education level, and no correlations with either SES or age. In this study, no correlation emerged between mothers’ RF and their education level (Spearman’s ρ = 0.063, *p* = 0.695).

This study’s first author coded all of the transcripts. A group of 10 randomly selected transcripts was re-coded by an independent rater. The inter-rater reliability was excellent (Cohen’s *k* = 0.87). Both coders were blinded to children’s scores on the measures of mentalization and mental-state talk.

#### Children’s Mentalization

Two measures were used to assess the mentalization ability of the children: (1) a mentalization test adapted from the measure designed by O’Connor and Hirsch (1999) and (2) the mental-state talk produced within an autobiographical narrative, the Child Attachment Interview (CAI). Two different measures were used to evaluate mentalization in an autobiographical narrative focused on attachment to parents as well as in an impersonal context because, according to Fonagy et al. (1998), reflective capacity in the attachment context should not be expected to generalize to other domains.

##### O’Connor and Hirsch’s (1999) measure of mentalization

A semi-structured interview was adapted from the measure developed by O’Connor and Hirsch (1999). This brief interview utilizes a photograph of a typical school setting in which a schoolteacher chooses a student from among different pupils who have their hands raised to answer her question. After explaining to the child that the scene takes place during a lesson, the child’s attention is drawn to one of the pupils who had not been chosen by the teacher. The child is then asked why, in his/her opinion, the pupil was not chosen; what the teacher may think and feel; what the pupil may think and feel; and what will happen afterward.

According to the guidelines suggested by O’Connor and Hirsch (1999), the narratives produced by the children were evaluated on three increasing levels of mentalization ability.

At level 1, references to mental states are lacking or refer to general attributions or stable character traits (e.g., “The teacher didn’t choose that pupil because she didn’t see him” or “The teacher didn’t choose that pupil because she shows favoritism to some other pupils”). Level 1 narratives lack references to specific events or situations. As indicated by O’Connor and Hirsch (1999, p. 261), “the behavior is not considered as influenced by the context, and there is no attempt to understand the circumstances that determine that behavior.”

Level 2 narratives include, at a moderate level of mentalization, specific references to thoughts and feelings that clearly indicate an ability to understand the relationships among behavior, thoughts, and internal states. A level 2 narrative shows awareness that specific internal experiences are linked to a specific external context (e.g., “The teacher didn’t choose that pupil because she knows that he knows the right answer and wants to give other pupils the opportunity to talk”).

Level 3 narratives are characterized by a more advanced level of mentalization and indicate second-order mentalizing abilities. At this level, the presence of recursive thinking or of a more advanced ability to reflect on mental states can be observed (e.g., “The teacher didn’t choose that pupil and chose the girl because she realizes that the girl is very sad and always remains aside, and the teacher wants to show her some consideration”).

No previous study has shown any significant correlation between O’Connor and Hirsch’s (1999) measure and age, gender, verbal IQ, or verbal fluency. Significant positive correlations have been found with measures of adjustment and friendship quality while inverse correlations emerged with a measure of depressive symptoms (O’Connor and Hirsch, 1999).

The second author, blinded to the coding of the psychological lexicon and of maternal RF and attachment patterns, coded the mentalization measure adapted from O’Connor and Hirsch (1999). An independent coder coded half of the tests. The inter-rater reliability was excellent (Cohen’s *k* = 0.82).

##### Child Attachment Interview (CAI)

The CAI (Shmueli-Goetz et al., 2000) is a semi-structured interview designed to assess the child’s state of mind regarding the attachment to each parent in middle childhood and adolescence. The CAI protocol consists of 17 questions regarding the family composition and how the child describes him/herself and his/her relationship with each parent. The child is asked to talk about specific relational episodes with each parent, specifically regarding when he/she is ill, upset, or angry, feels neglected or rejected, or needs help. Like the AAI, the CAI investigates emotional responses to loss and separation experiences.

For the purposes of this study, CAIs were not used to classify attachment patterns for that had been examined in another study. In the current study, CAIs were used to evaluate the frequency and quality of the mental-state talk in the context of an autobiographical narrative. A mastery of words referring to inner states, such as beliefs, emotions, and desires, implies that the child understands that human beings may have different psychological states. It also shows that the child is capable of representing these mental states and using them to understand the behavior of others (for reviews, see Carpendale and Lewis, 2004; Symons, 2004).

For this reason, the frequency of mental state terms has been considered a good index of psychological understanding and an important marker of children’s mental state awareness in real-life contexts (Bartsch and Wellman, 1995). Furthermore, as Peterson and Slaughter (2006) noted, the analysis of mental state words in narratives allows the exploration of individual differences in the use of ToM capacities, especially when the False–Belief test is no longer informative.

Mental-state talk in the CAI transcripts was coded into the following categories:

• Emotional terms: every term that refers to one’s or others’ emotional state (e.g., “Every time we met, we **had a quarrel**”; “I **like** company of friends”);
• Cognitive terms: every term that refers to a cognitive process (e.g., “It is hard to **understand**”; “I can’t **remember** what I did that time”);
• Volitional terms: every term that refers to need, desire or preference (e.g., “I never **want** to be on the wrong side”; “I **needed** some help”);
• Terms related to skills: every term that refers to one’s or others’ skill (e.g., “She was very **good at** persuading people”; “When I don’t **succeed** in doing maths, he always helps me”).

Uncertainty markers included uncertain mental verbs (e.g., believe, suppose), modal adverbs (e.g., perhaps), modal verbs (e.g., should), and modal adjectives (e.g., probable, possible, and likely), (e.g., “Maybe now it’s different”; “It’s just because she is not sure, just for this reason I suppose”). References to terms that indicate uncertainty can signal one’s awareness of the opacity of mental states and can be regarded as an indicator of mentalization ability (Howard et al., 2008).

The following data were extracted from the CAI transcripts: total number of words produced (verbal productivity), numbers of emotional, cognitive, and volitional lexicon terms, number of ability-related terms, number of uncertainty markers, and the sum of terms referring to mental states (overall psychological lexicon).

Two independent coders, who had been appropriately trained, evaluated all of the CAIs with respect to the frequency of use and quality of the mental-state talk. They were blinded to maternal RF and attachment patterns. The inter-rater reliability was excellent (Cohen’s *k* = 0.86).

To analyze the richness of the psychological lexicon, the root types/token ratio (types/√tokens) was calculated for the total number of terms referring to mental states and for the specific categories of the emotional, cognitive, and volitional lexicons as well as of words related to skills. The square root has a mitigating effect on the impact of the number of tokens; this transformation is necessary because the number of types increases much more slowly than does the number of tokens in sampling texts (Rizzi, 1995).

#### Children’s Verbal Intelligence

The WISC-III verbal scale was administered to evaluate children’s verbal IQ.

### Procedure

The headmaster of the school sent an information letter about the research project to 360 families of children attending the second year of the middle school. Of the 360 families contacted, 46 mothers (13%) agreed to be contacted further, and 41 ultimately agreed to participate in the study. Both children and parents were informed about the aim of the study, after which both parents gave their written consent to participate. Consent to administer the WISC-III verbal scale was obtained only for 24 children. All evaluations took place at the school. Senior psychology students who had previously been trained in the administration of the instruments administered the measures. The AAIs were audiotaped, the CAIs were videotaped, and then both were transcribed verbatim.

This study followed the APA ethical guidelines (American Psychological Association, 2010).

## Results

### Maternal Reflective Functioning and Maternal Attachment Pattern

**Table 1** reports the descriptive statistics of the mothers’ scores on the RFS and the AAI subscales related to the state of mind. Correlation analyses revealed no significant associations between RF variables and maternal education (*r*’s between –0.12 and 0.23).

**Table 1:** Descriptive statistics of mothers’ scores on Reflective Functioning (RF) and Adult Attachment Interview (AAI) subscales.

	Minimum	Maximum	*M*	SD
**RF**				
RF global	-1	7	3.71	1.6
Marker A	0	4	0.83	1.07
Marker B	0	16	5.41	4.27
Marker C	0	10	1.73	2.32
Marker D	0	1	0.02	0.16
Ref. pos. MS	0	6	1.34	1.6
Ref. neg. MS	0	10	4.05	2.79
Ref. mix. MS	0	9	1.15	2.24
**AAI scales**				
Idealization regarding mother	1	7	2.63	1.63
Idealization regarding father	1	6	2.38	1.54
Derogation regarding mother	1	8	1.79	1.78
Derogation regarding father	1	9	1.46	1.52
Overall derogation of attachment	1	9	2.07	1.93
Insistence on lack of recall	1	5	1.61	0.95
Involving anger regarding mother	1	9	2.12	2.28
Involving anger regarding father	1	8	1.61	1.64
Passivity of thought processes	1	8	2.94	1.90
Coherence of the transcript	3	8.5	5.73	1.56
Coherence of mind	3	8.5	5.73	1.56

*Marker A, Awareness of the nature of mental states; Marker B, Explicit effort to tease out the mental states underlying behavior; Marker C, Recognizing developmental aspects of mental states; Marker D, Mental states in relation to the interviewer; Ref. pos. MS, references to positive mental states; Ref. neg. MS, Reference to negative mental states; Ref. mix MS, Reference to mixed-ambivalent mental states.*

The distribution of mothers’ attachment classifications in our sample was as follows: 25 Secure (61%), 6 Dismissive (14.6%), 6 Preoccupied (14.6%), 2 Cannot Classify (4.8%), and 2 Unresolved (4.8%). Because of the small number of mothers classified as Dismissive, Preoccupied, Cannot Classify, and Unresolved, these groups were combined into a single insecure group. Therefore, the final distribution of attachment classifications was as follows: 25 secure (61%) and 16 (39%) insecure. Analysis of variance revealed no significant differences in maternal education as a function of attachment security (*F* = 0.976). Correlation analyses revealed no significant associations between scores on the state-of-mind subscales and education (*r*’s between –0.13 and 0.24).

Mann–Whitney *U* comparisons revealed significant differences in RF variables between secure and insecure mothers (**Table 2**). Specifically, securely attached mothers reported higher overall RF scores in addition to higher scores on all of the other markers of RF except for marker D (“Mental states in relation to interviewer”). They also reported more frequent references both to positive and negative mental states. No significant difference emerged in the frequencies of references to mixed-ambivalent mental states as a function of attachment security.

**Table 2:** Comparisons between insecure and secure mothers on RF scores.

	Insecure mothers *N* = 16		Secure mothers *N* = 25			
	*M*	SD	*M*	SD	*Z*	*p*
RF global	2.56	1.21	4.44	1.39	-3.741	<0.0001
Marker A	0.38	0.72	1.12	1.17	-2.228	0.026
Marker B	2.94	3.42	7.00	4.04	-3.382	0.001
Marker C	0.75	1.00	2.36	2.71	-2.120	0.034
Marker D	0	0	0.04	0.20	-.800	n.s.
Ref. pos. MS	0.31	0.48	2	1.73	-3.556	<0.0001
Ref. neg. MS	2.69	2.44	4.92	2.69	-2.542	0.011
Ref. mix. MS	0.69	1.70	1.44	2.52	-1.345	n.s.

*Marker A, Awareness of the nature of mental states; Marker B, Explicit effort to tease out the mental states underlying behavior; Marker C, Recognizing developmental aspects of mental states; Marker D, Mental states in relation to the interviewer; Ref. pos. MS, references to positive mental states; Ref. neg. MS, Reference to negative mental states; Ref. mix MS, Reference to mixed-ambivalent mental states.*

Correlations analyses revealed significant associations between RF variables and scores on AAI state-of-mind subscales (**Table 3**). In particular, the Coherence subscale of the transcript (CT) correlated positively with most RF variables except for the marker *D* score and references to mixed-ambivalent mental states. With regard to the other AAI subscales, Overall derogation of attachment (OD) and Insistence on lack of recall (ILR) showed a more consistent pattern of significant association with different RF variables than did the other scales. The marker *D* score and references to mixed-ambivalent mental states did not correlate with any AAI subscales.

**Table 3:** Spearman correlation coefficients between maternal scores on the RF and AAI subscales.

	IDM	IDF	DM	DF	OD	ILR	IAM	IAF	PTP	CT
RF global	-0.27	-0.33^∗^	-0.33^∗^	-0.19	-0.39^∗^	-0.52^∗∗∗^	-0.20	-0.08	-0.31^∗^	0.76^∗∗∗^
Marker A	-0.07	-0.01	-0.24	-0.18	-0.44^∗^	-0.29	-0.33^∗^	-0.27	-0.09	0.46^∗∗^
Marker B	-0.33^∗^	-0.24	-0.28	-0.22	-0.45^∗∗^	-0.51^∗∗∗^	-0.17	-0.14	-0.15	0.61^∗∗∗^
Marker C	-0.06	-0.14	-0.21	-0.12	-0.20	-0.18	-0.28	-0.01	-0.40^∗∗^	0.49^∗∗^
Marker D	-0.17	-0.17	-0.08	-0.06	-0.11	-0.12	-0.09	-0.07	-0.20	0.20
Ref. pos. MS	-0.10	-0.09	-0.40^∗∗^	-0.27	-0.47^∗∗^	-0.30	-0.34^∗^	-0.34^∗^	-0.24	0.53^∗∗^
Ref. neg. MS	-0.35^∗^	-0.37^∗^	-0.17	-0.09	-0.26	0.46^∗∗^	-0.15	-0.07	-0.12	0.52^∗∗^
Ref. mix. MS	-0.15	-0.16	-0.18	-0.12	-0.25	-0.24	-0.18	-0.02	-0.16	0.30

*Marker A, Awareness of the nature of mental states; Marker B, Explicit effort to tease out the mental states underlying behavior; Marker C, Recognizing developmental aspects of mental states; Marker D, Mental states in relation to the interviewer; Ref. pos. MS, references to positive mental states; Ref. neg. MS, Reference to negative mental states; Ref. mix MS, Reference to mixed-ambivalent mental states; IDM, Idealizing mother; IDF, Idealizing father; DM, Derogation mother; DF, Derogation father; OD, Overall Derogation; ILR, Insistence on lack of recall; IAM, Involving anger mother; IAF, Involving Anger Father; PTP, Passivity of thought processes; CT, Coherence of the transcript; ^∗^p < 0.05, ^∗∗^p < 0.01, ^∗∗∗^p < 0.001.*

### Children’s Mentalization and Mental-State Talk

Based on the test adapted from O’Connor and Hirsch (1999) 11 children (26.8%) showed a low level of mentalization while 24 children (58.5%) showed ordinary and six children (14.6%) showed high levels of mentalization. Analyses of variance revealed no significant differences in mothers’ education (*df* = 2; *F* = 0.641) and children’s verbal IQ. (*df* = 2; *F* = 1.502) as a function of children mentalization. Chi-square analysis revealed no significant differences between boys and girls in levels of mentalization (χ^2^ = 0.35; *df* = 1; *p* = 0.55).

Regarding the children’s mental-state talk in the CAI narratives, correlation analyses revealed no significant associations with either maternal education (*r*’s between –0.064 and 0.25) or children’s verbal IQ. (*r*’s ranged from –0.027 to 0.16). A higher frequency in the use of markers of uncertainty was found in girls (*F* = 8.304; *p* = 0.006). No other significant gender differences emerged regarding the use of mental-state talk.

We also calculated Spearman’s correlations between the mentalization measure adapted from O’Connor and Hirsch (1999) and the different categories of the mental-state lexicon (**Table 4**). Analyses showed that children’s level of mentalization correlated positively with the emotional, cognitive, and volitional lexicons, as well as with the overall psychological lexicon. Markers of uncertainty were positively, though not significantly, associated with level of mentalization.

**Table 4:** Spearman correlation coefficients between the mentalization measure (adapted from O’Connor and Hirsch, 1999) and children’s mental-state talk.

	Mentalization
Emotional lexicon	0.38^∗^
Cognitive lexicon	0.41^∗∗^
Volitional lexicon	0.38^∗^
Lexicon referred to abilities	0.29
Uncertainty markers	0.29
Overall psychological lexicon	0.51^∗∗∗^

*^∗^p < 0.05, ^∗∗^p < 0.01, ^∗∗∗^p < 0.001.*

### Maternal Reflective Functioning and Children’s Mental-State Talk and Level of Mentalization

Spearman’s correlations between maternal RF scores and children’s mental-state talk are reported in **Table 5**. The overall maternal RF score correlated positively with the cognitive, volitional and overall psychological lexicons and with uncertainty markers in children’s narratives.

**Table 5:** Spearman correlation coefficients between maternal scores on the RF scale, scores from the children’s mentalization measure (adapted from O’Connor and Hirsch, 1999) and children’s mental-state talk.

	Emotional lexicon	Cognitive lexicon	Volitional lexicon	Lexicon referred to abilities	Uncertainty markers	Overall psychological lexicon
RF global	0.23	0.38^∗^	0.45^∗∗^	0.21	0.51^∗∗∗^	0.42^∗∗^
Marker A	0.32^∗^	0.35^∗^	0.21	-0.02	0.25	0.38^∗^
Marker B	0.12	0.37^∗^	0.43^∗∗^	0.16	0.54^∗∗∗^	0.36^∗^
Marker C	0.12	0.29	0.23	0.18	0.30	0.26
Marker D	0.01	-0.25	-0.15	-0.09	-0.16	-0.17
Ref. pos. MS	0.04	0.20	0.22	0.02	0.23	0.25
Ref. neg. MS	0.09	0.38^∗^	0.27	0.07	0.38^∗^	0.31^∗^
Ref. mix. MS	0.35^∗^	0.29	0.30	0.26	0.44^∗∗^	0.37^∗^

*Marker A, Awareness of the nature of mental states; Marker B, Explicit effort to tease out the mental states underlying behavior; Marker C, Recognizing developmental aspects of mental states; Marker D, Mental states in relation to the interviewer; Ref. pos. MS, references to positive mental states; Ref. neg. MS, Reference to negative mental states; Ref. mix MS, Reference to mixed-ambivalent mental states. ^∗^p < 0.05, ^∗∗^p < 0.01, ^∗∗∗^p < 0.001.*

When considering the specific RF markers, neither marker C nor marker D correlated with any mental state talk category in children’s narratives whereas marker A (Awareness of the nature of mental states) and marker B (Explicit effort to tease out mental states underlying behavior) were significantly associated with different categories of children’s mental state talk.

Maternal references to negative mental states correlated with the cognitive lexicon, the overall psychological lexicon and uncertainty markers. Similarly, maternal references to mixed-ambivalent mental states significantly correlated with the emotional lexicon, the overall psychological lexicon and uncertainty markers. No significant correlation was found between maternal references to positive mental states and children’s mental-state talk.

The children’s mentalization level correlated positively with the mothers’ overall RF score (ρ = 0.32; *p* < 0.05) and references to mixed-ambivalent mental states (ρ = 0.42; *p* < 0.01). No significant correlations were found with any of the four markers of reflecting functioning and with references to positive or negative mental-states.

### Maternal Attachment and Children’s Mental-State Talk and Level of Mentalization

With regard to the relationship between maternal attachment pattern and mental state talk, analyses of variance revealed no significant differences between the children of secure and insecure mothers in terms of the frequency of use of mental-state terms (*F*s between 0.006 and 0.822). To explore whether a dimensional perspective of attachment security yielded a significant relationship, a Pearson’s correlation analysis was performed between the different categories of children’s mental-state talk and the mother’s scores on the AAI subscales. An inverse correlation emerged between the frequency of use of markers of uncertainty in the children narratives and two scales of the AAI transcript, namely, maternal idealization regarding the mother (*r* = –0.435; *p* = 0.005) and maternal insistence on lack of recall (*r* = –0.337; *p* = 0.031).

No significant associations emerged between maternal attachment security and children’s level of mentalization (χ^2^ = 4.49; *p* = 0.105). Nevertheless, all six children with a level of mentalization of three were children of secure mothers whereas about half of the children who obtained a score of one or two on the O’Connor and Hirsch (1999) measure had mothers with an insecure attachment (45.5% for children who obtained a score of 1 and 45.8% for children who obtained a score of 2). When collapsing categories one and two together, a significant relationship between maternal attachment security and children’s level of mentalization was found (χ^2^ = 4.49; *p* = 0.034). A Spearman’s correlation analysis between children’s mentalization level and mothers’ scores on the AAI subscales was also carried out. No significant correlations emerged (*r*’s between –0.18 and 0.16).

To evaluate the extent to which maternal variables could predict children’s use of mental terms, we conducted a stepwise regression analysis using maternal RF global score and the classification of secure/insecure from the AAI as predictors of both children’s overall psychological lexicon and markers of uncertainty. The final models are reported in **Table 6**. The models explain approximately 11% of the variance in children’s psychological lexicon and markers of uncertainty. Specifically, only maternal RF predicted children’s use of mental terms (*t* = 2.19, *p* = 0.034 for the overall psychological lexicon; *t* = 2.16, *p* = 0.037 for markers of uncertainty).

**Table 6:** Relationships between maternal variables and children’s production of mental-state terms.

	Children’s overall psychological lexicon *F*(1,39) = 4.828; *R*^2^= 0.11; *p* = 0.034					Children’s markers of uncertainty *F*(1,39) = 4.674; *R*^2^= 0.11; *p* = 0.037				
	*B*	SE	β	*t*	*p*	*B*	SE	β	*t*	*p*
Maternal RF global	0.134	0.061	0.332	2.19	0.034	0.061	0.028	0.327	2.16	0.037

## Discussion

### Maternal Reflective Functioning and Children’s Mentalization

Analyses of maternal RF yielded significant correlations with children’s mentalization and all subtypes of mental-state talk with the exception of the emotional lexicon. The category of mental-state talk that was most significantly connected to maternal mentalization was the uncertainty markers, followed in order by the volitional lexicon, overall psychological lexicon and cognitive lexicon. Therefore, it appears that the predisposition to use the language of emotional states *tout court* is less distinctive of mentalization ability than the tendency to use a mentalistic lexicon that refers to oneself and others in terms of psychological agents having their own beliefs, wishes and thoughts.

Our results are consistent with the hypothesis that the cognitive lexicon is an indicator of mentalization ability, especially when it expresses a degree of uncertainty. Developmental psychologists have long observed that a child’s ability to distinguish between different degrees of uncertainty is a sophisticated competence that is reached later in development. Some researchers consider these cognitive terms to be the only genuine category within the psychological lexicon (Moore et al., 1989). The degree of uncertainty expressed in linguistic production appears to be strictly connected to the RF indicator described by Fonagy et al. (1998) as the awareness that mental states are opaque and susceptible to being masked. To express such awareness, an individual must use uncertainty markers, including uncertain mental verbs, adjectives, adverbs, and modal verbs.

Our results are also consistent with previous studies showing that a mother’s mentalization ability predicts the same ability in her children (Meins et al., 1998, 2002, 2003; Steele et al., 2008). However, such comparisons between studies should be considered with caution, given their differences in measurement tools and in the ages of the children sampled between this report and previous studies.

The current study also aimed to identify which specific aspects of maternal RF are most strongly related to children’s mentalization ability and mental-state talk, with the purpose of understanding which maternal competencies should represent the main focus of therapeutic actions. Marker A (awareness of the nature of mental states) and, especially, marker B (explicit effort to highlight the mental states that lay behind behavior) were the most significant maternal competencies.

Children’s mentalization abilities and their mental-state talk were more correlated with maternal competence in the mentalization of negative or mixed-ambivalent mental states than it was with maternal competence in the mentalization of positive mental states, which is consistent with results from previous studies (Dunn and Brown, 2001; Hughes and Dunn, 2002; Sharp et al., 2006; Steele et al., 2008).

### Maternal Attachment and Children’s Mentalization

Although no significant differences were observed between the mentalization abilities of children of secure vs. insecure mothers, all children with a high level of mentalization were the children of secure mothers. Using a dimensional perspective of attachment, it was found that a maternal dismissive strategy, based on idealization and insistence on a lack of recall, was significantly and negatively correlated with a marker of children’s mentalization ability (i.e., frequency of uncertainty markers in linguistic production). Future studies should focus on the specific impact of this maternal strategy on children’s mentalization abilities. Specific difficulties in recognizing mixed and negative emotions have been detected in the children of dismissive mothers (Steele et al., 2008).

The results of this study could have been influenced by the distribution of insecure attachment patterns among the mothers included in our sample. In particular, Steele et al. (1999, 2008) observed that dismissive mothers represented 70% of insecure mothers, whereas only 38% of insecure mothers in our sample were dismissive. Another possible interpretation of the absence of an association between the security of maternal attachment and children’s mentalization is the older age of the children in our sample compared to that of previous studies (i.e., preadolescent vs. primarily preschool-aged children). This result was consistent with the findings of a longitudinal study by Steele et al. (2008), who observed a reduction in the effect of the mothers’ attachment on the understanding of emotions in their 11-year-old children compared to their 6-year-old children.

### Influence of the Constructs and Instruments on Findings in this Study

The differences between this study and previous reports, related to the operationalization of the constructs and the measures, permit only a tentative comparison. Some studies have examined younger children with ToM tests and have mainly operationalized maternal mentalization as maternal mind-mindedness (Meins et al., 1998, 2002, 2003). We preferred not to use ToM tests in this study to evaluate mentalization in children because many studies (e.g., Bloom and German, 2000) have observed that verbal false–belief tests require linguistic and cognitive abilities that do not specifically grasp all the dimensions of “mentalization” (i.e., relational and affective regulation aspects). Interestingly, this study did not find significant correlations between maternal RF and education, nor between children’s mentalization and mental-state talk, that in turn did not also correlate with children’s verbal IQ.

Previous studies that also evaluated children’s mentalization using the psychological lexicon produced in an autobiographical interview have noted that the frequency of a psychological lexicon in a real-life context is a good indicator of one’s awareness of mental states. Moreover, these studies have indicated that such an approach additionally allows the exploration of individual differences in mentalization (Peterson and Slaughter, 2006) and of the influences of maternal mentalization on children’s mentalization (Ruffman et al., 2002; Taumoepeau and Ruffman, 2006, 2008; Mcquaid et al., 2008).

Sharp and Fonagy (2008) highlighted the confusion between the many concepts of maternal RF derived from different theoretical backgrounds and between the different instruments or measurement tools used to study these concepts. Maternal RF has only been used in studies by Fonagy, with the aim of studying the impact of RF on the performance of 5- to 11-year-old children on ToM tests (Fonagy et al., 1997) and on tests of understanding emotions (Steele et al., 1999, 2002, 2003). However, RFS has shown predictive validity (Fonagy et al., 1998), supporting its use in this application in the present study. However, our study would have been more complete had we also used the parental RF-PDI (Parent Developmental Interview; Slade et al., 2004), which measures the maternal ability to treat a specific child as a psychological agent. In fact, it has been observed that parents manifested different degrees of mentalization abilities relative to different children (Sharp and Fonagy, 2008). Moreover, AAI and RFS are not independent measures. On one hand, this may present a limitation of the present study, but on the other hand, it was interesting to explore how different insecure patterns may impact RF. Following a dimensional approach to AAI, the current study found that involving anger, a marker of the preoccupied state of the mind regarding attachment, was inversely correlated with RF in the specific domain of positive mental state, while the two different dismissing strategies – i.e., derogation and idealization – correlated with specific impairment of RF. Idealizing mothers resulted not able to mentalize negative mental states, while derogating mothers showed a specific deficit in mentalizing positive mental states.

Concerning the evaluation of mentalization in preadolescents, we chose to use one assessment measure within the autobiographical personal context and one within a non-personal context. Interestingly, the two measures were significantly correlated and neither showed a significant effect with gender, IQ, or maternal education.

### Study Limitations

In addition to some of the limitations mentioned above, another limitation of the current study was that the frequency of the psychological lexicon, unlike RFS and the test adapted by O’Connor and Hirsch (1999), did not consider the possible distorted quality of mentalization. Recent studies revealed this aspect to be crucial in the evaluation of mentalization ability (Sharp et al., 2007).

Furthermore, less than 15% of the mothers originally contacted agreed to participate in the research. Thus, it is not possible to know the extent to which our sample can be generalizable and what implications this limited participation might have had on the present study. Compared to other previous non-clinical samples (Bakermans-Kranenburg and van IJzendoorn, 2009), the dismissive classification in this study appeared to be underrepresented in the AAI. In addition, the study could have been methodologically improved if an on-line measure of the mother-child interaction had been used and if verbal IQ had been obtained for all of the children.

## Conclusion

Children’s mentalization was positively correlated with maternal RF, particularly the ability to mentalize negative or mixed-ambivalent mental states. These results are in line with studies indicating a *trans*-generational continuity of mentalizing abilities between mothers and their children. Significant differences in mentalization abilities between children of secure and insecure mothers were not observed, although all children with a high level of mentalization had secure mothers. Future studies should investigate the long-term impact of maternal RF on children’s mentalization abilities, especially during adolescence, when a mother’s ability to understand her child’s mental states could be an important protective factor against the development of psychopathological conditions or health-risk behaviors. The findings of the present study support the need to implement mentalization-based treatments for parents of pre-adolescents and to investigate their outcomes.

## Conflict of Interest Statement

The authors declare that the research was conducted in the absence of any commercial or financial relationships that could be construed as a potential conflict of interest.
